# Supplementary material for: Ciguatera-Causing Dinoflagellate Gambierdiscus spp. (Dinophyceae) in a Subtropical Region of North Atlantic Ocean (Canary Islands): Morphological Characterization and Biogeography
Source: Toxins (Basel). 2019 Jul 19;11(7):423. doi: 10.3390/toxins11070423 (PMC6669716; doi:10.3390/toxins11070423)
Supplement: Supplementary file 1 [file toxins-11-00423-s001.pdf]

Table S1. Cell concentrations (cells·mL^{-1}) of *Gambierdiscus* and others epiphytic dinoflagellate genus on the islands of La Palma and La Gomera (Canary archipelago).

Island	Station	Gamgierdiscus	Prororocentrum	Coolia	Sinophysis	Ostreopsis	Vulcanodinium	Heterocapsa	Scrippsiella
La Palma	Charco azul	0	0	0	0	596	0	0	0
La Palma	Charco azul	0	0	0	0	447	0	0	0
La Palma	Charco azul	0	0	0	0	0	0	0	0
La Palma	Charco azul	0	0	0	0	128	0	0	0
La Palma	Charco azul	0	0	0	0	0	0	0	0
La Palma	Los Cancajos	0	0	0	0	0	0	0	0
La Palma	Los Cancajos	0	0	0	0	0	922	0	0
La Palma	Los Cancajos	0	0	0	0	62	62	0	0
La Palma	Los Cancajos	0	0	133	0	0	266	0	0
La Palma	Los Cancajos	0	0	0	0	940	313	0	0
La Palma	Tazacorte beach	0	0	0	0	1340	223	1340	0
La Palma	Tazacorte beach	29	0	0	0	783	196	391	0
La Palma	Tazacorte beach	0	0	0	0	2759	0	690	345
La Palma	Tazacorte beach	0	0	0	0	265	0	1191	0
La Palma	Tazacorte beach	0	0	0	0	957	0	638	160
La Palma	Charco verde	0	0	0	0	511	340	0	0
La Palma	Charco verde	0	0	0	0	348	87	0	0
La Palma	Charco verde	0	0	613	0	1532	306	0	0
La Palma	Charco verde	21	0	0	0	1702	425	0	0
La Palma	Charco verde	0	0	0	425	1702	0	0	0
La Palma	Charco verde	0	0	0	0	0	0	0	0
La Palma	La Zamora beach	0	0	56	0	113	0	0	0
La Palma	La Zamora beach	0	0	0	0	0	0	0	92
La Palma	La Zamora beach	0	75	0	0	375	0	0	0
La Palma	La Zamora beach	8	170	0	0	0	0	0	0
La Gomera	Santiago beach	0	0	0	0	0	0	0	0
La Gomera	Santiago beach	0	0	0	0	0	0	0	0
La Gomera	Santiago beach	83	241	241	0	0	482	0	0
La Gomera	Santiago beach	0	1659	0	0	0	0	0	0
La Gomera	Santiago beach	0	0	0	0	0	0	0	0
La Gomera	Santiago beach	0	672	1343	0	0	1343	0	0
La Gomera	Santiago beach	0	580	0	0	290	1450	0	0
La Gomera	Charco Condesa	0	0	213	0	851	0	0	0
La Gomera	Charco Condesa	6	239	0	0	239	0	0	0
La Gomera	Charco Condesa	0	183	0	183	0	0	0	0
La Gomera	Charco Condesa	16	0	0	0	1595	0	0	0
La Gomera	Charco Condesa	7	0	96	0	96	0	0	0
La Gomera	Charco Condesa	11	0	0	0	656	0	0	0
La Gomera	Charco Condesa	0	165	165	165	329	0	0	0
La Gomera	Charco Condesa	8	788	0	0	315	0	0	0
La Gomera	Charco Condesa	10	414	1035	207	0	0	0	0
La Gomera	Charco Condesa	17	0	0	0	348	0	0	0
La Gomera	La Cueva beach	8	79	0	0	0	79	159	0
La Gomera	La Cueva beach	17	0	0	0	0	0	0	0
La Gomera	La Cueva beach	0	0	358	0	0	179	0	0
La Gomera	La Cueva beach	6	0	0	66	66	66	0	0
La Gomera	La Cueva beach	28	0	574	574	574	2872	0	0
La Gomera	La Cueva beach	14	5672	0	567	567	1418	0	0
La Gomera	La Cueva beach	0	0	0	0	0	277	0	0
La Gomera	La Cueva beach	6	0	123	123	123	245	0	0
La Gomera	La Cueva beach	11	0	0	0	0	228	0	0
La Gomera	La Cueva beach	12	159	79	79	40	40	0	0
La Gomera	La Cueva beach	88	540	180	180	0	719	0	0

Table S2. Mean rank values in Kruskal-Wallis test for cell size measurements and morphological parameters (see material and methods) of *Gambierdiscus* species.

Parameter	Species	N	Mean Rank
D (cell depth)	G. australes	94	256,86
	G. caribaeus	72	193,59
	G. carolinianus	72	163,66
	G. excentricus	81	334,19
	G. silvae	88	69,23
	Total	407	
W (cell width)	G. australes	94	249,63
	G. caribaeus	71	217,18
	G. carolinianus	72	167,40
	G. excentricus	80	312,02
	G. silvae	88	71,77
	Total	405	
R1	G. australes	92	244,08
	G. caribaeus	77	311,51
	G. carolinianus	63	80,25
	G. excentricus	78	206,92
	G. silvae	88	133,68
	Total	398	
R2	G. australes	85	247,94
	G. caribaeus	77	146,21
	G. carolinianus	65	177,03
	G. excentricus	77	349,79
	G. silvae	84	55,86
	Total	388	
R3	G. australes	88	329,63
	G. caribaeus	83	144,51
	G. carolinianus	64	77,55
	G. excentricus	77	275,82
	G. silvae	82	129,43
	Total	394	

Table S3. Pairwise distances (773 nt alignment) between 54 Gambierdiscus sequences obtained in this study, calculated as the proportion (p) of nucleotide sites at which two sequences being compared are different. It was obtained by dividing the number of nucleotide differences by the total number of nucleotides compared, computed as d: Transitions + Transversions: proportion of nucleotide sites that are different (MEGA7 software). Reference sequences for G. silvae, G.

#	*G. silvae*	Acc. No.	Island (Station)	*G. silvae* (Acc.No. GU968518)																																																							
1	VGO1355	MK649614	La Gomera (Char	0,001																																																							
2	VGO1358	MK649615	La Gomera (Char	0,000	0,001																																																						
3	VGO1363	MK649616	La Gomera (Char	0,000	0,001	0,000																																																					
4	VGO1368	MK649620	La Gomera (Char	0,000	0,001	0,000	0,000																																																				
5	VGO1376	MK649618	La Gomera (Santi	0,000	0,001	0,000	0,000	0,000																																																			
6	VGO1378	MK649619	La Gomera (Char	0,000	0,001	0,000	0,000	0,000	0,000																																																		
7	VGO1379	MK649617	La Gomera (Char	0,000	0,001	0,000	0,000	0,000	0,000	0,000																																																	
	G. excentricus											*G. excentricus* (Acc.No. JF303073)																																															
8	PL9B4	MK649631	La Gomera (La C	0,113	0,114	0,113	0,113	0,113	0,113	0,113	0,113	0,000																																															
9	PL9B5	MK649623	La Gomera (La C	0,113	0,114	0,113	0,113	0,113	0,113	0,113	0,113	0,000	0,000																																														
10	PL9C6	MK649628	La Gomera (Char	0,113	0,114	0,113	0,113	0,113	0,113	0,113	0,113	0,000	0,000	0,000																																													
11	PL11C3	MK649627	La Gomera (Santi	0,113	0,114	0,113	0,113	0,113	0,113	0,113	0,113	0,000	0,000	0,000	0,000																																												
12	PL11D4	MK649626	La Palma (La Zan	0,113	0,114	0,113	0,113	0,113	0,113	0,113	0,113	0,000	0,000	0,000	0,000	0,000																																											
13	PL11C2	MK649645	La Gomera (Santi	0,113	0,114	0,113	0,113	0,113	0,113	0,113	0,113	0,000	0,000	0,000	0,000	0,000	0,000																																										
14	PL11C6	MK649632	La Gomera (Char	0,113	0,114	0,113	0,113	0,113	0,113	0,113	0,113	0,000	0,000	0,000	0,000	0,000	0,000	0,000																																									
15	PL14E3	MK649647	La Gomera (La C	0,113	0,114	0,113	0,113	0,113	0,113	0,113	0,113	0,000	0,000	0,000	0,000	0,000	0,000	0,000	0,000																																								
16	PL15C6	MK649649	La Gomera (La C	0,113	0,114	0,113	0,113	0,113	0,113	0,113	0,113	0,000	0,000	0,000	0,000	0,000	0,000	0,000	0,000	0,000																																							
17	PL15D2	MK649648	La Gomera (La C	0,113	0,114	0,113	0,113	0,113	0,113	0,113	0,113	0,000	0,000	0,000	0,000	0,000	0,000	0,000	0,000	0,000	0,000																																						
18	**VGO1357**	MK649629	La Gomera (Char	0,113	0,114	0,113	0,113	0,113	0,113	0,113	0,113	0,000	0,000	0,000	0,000	0,000	0,000	0,000	0,000	0,000	0,000	0,000																																					
19	**VGO1359**	MK649630	La Gomera (Char	0,113	0,114	0,113	0,113	0,113	0,113	0,113	0,113	0,000	0,000	0,000	0,000	0,000	0,000	0,000	0,000	0,000	0,000	0,000	0,000																																				
20	**VGO1361**	MK649634	La Gomera (Char	0,113	0,114	0,113	0,113	0,113	0,113	0,113	0,113	0,000	0,000	0,000	0,000	0,000	0,000	0,000	0,000	0,000	0,000	0,000	0,000	0,000																																			
21	**VGO1372**	MK649633	La Gomera (Char	0,113	0,114	0,113	0,113	0,113	0,113	0,113	0,113	0,000	0,000	0,000	0,000	0,000	0,000	0,000	0,000	0,000	0,000	0,000	0,000	0,000	0,000																																		
22	**VGO1373**	MK649635	La Palma (La Zan	0,113	0,114	0,113	0,113	0,113	0,113	0,113	0,113	0,000	0,000	0,000	0,000	0,000	0,000	0,000	0,000	0,000	0,000	0,000	0,000	0,000	0,000	0,000																																	
23	**VGO1374**	MK649642	La Gomera (Char	0,113	0,114	0,113	0,113	0,113	0,113	0,113	0,113	0,000	0,000	0,000	0,000	0,000	0,000	0,000	0,000	0,000	0,000	0,000	0,000	0,000	0,000	0,000	0,000																																
24	**VGO1380**	MK649639	La Gomera (La C	0,113	0,114	0,113	0,113	0,113	0,113	0,113	0,113	0,000	0,000	0,000	0,000	0,000	0,000	0,000	0,000	0,000	0,000	0,000	0,000	0,000	0,000	0,000	0,000	0,000																															
25	**VGO1381**	MK649638	La Gomera (La C	0,113	0,114	0,113	0,113	0,113	0,113	0,113	0,113	0,000	0,000	0,000	0,000	0,000	0,000	0,000	0,000	0,000	0,000	0,000	0,000	0,000	0,000	0,000	0,000	0,000	0,000																														
26	**VGO1383**	MK649640	La Gomera (Char	0,113	0,114	0,113	0,113	0,113	0,113	0,113	0,113	0,000	0,000	0,000	0,000	0,000	0,000	0,000	0,000	0,000	0,000	0,000	0,000	0,000	0,000	0,000	0,000	0,000	0,000	0,000																													
27	**VGO1384**	MK649641	La Gomera (Char	0,113	0,114	0,113	0,113	0,113	0,113	0,113	0,113	0,000	0,000	0,000	0,000	0,000	0,000	0,000	0,000	0,000	0,000	0,000	0,000	0,000	0,000	0,000	0,000	0,000	0,000	0,000	0,000																												
28	**VGO1386**	MK649646	La Gomera (La C	0,113	0,114	0,113	0,113	0,113	0,113	0,113	0,113	0,000	0,000	0,000	0,000	0,000	0,000	0,000	0,000	0,000	0,000	0,000	0,000	0,000	0,000	0,000	0,000	0,000	0,000	0,000	0,000	0,000																											
29	**VGO1387**	MK649636	La Palma (La Zan	0,113	0,114	0,113	0,113	0,113	0,113	0,113	0,113	0,000	0,000	0,000	0,000	0,000	0,000	0,000	0,000	0,000	0,000	0,000	0,000	0,000	0,000	0,000	0,000	0,000	0,000	0,000	0,000	0,000	0,000																										
30	**VGO1388**	MK649643	La Gomera (La C	0,113	0,114	0,113	0,113	0,113	0,113	0,113	0,113	0,000	0,000	0,000	0,000	0,000	0,000	0,000	0,000	0,000	0,000	0,000	0,000	0,000	0,000	0,000	0,000	0,000	0,000	0,000	0,000	0,000	0,000	0,000																									
31	**VGO1389**	MK649644	La Gomera (La C	0,113	0,114	0,113	0,113	0,113	0,113	0,113	0,113	0,000	0,000	0,000	0,000	0,000	0,000	0,000	0,000	0,000	0,000	0,000	0,000	0,000	0,000	0,000	0,000	0,000	0,000	0,000	0,000	0,000	0,000	0,000	0,000																								
32	**VGO1390**	MK649637	La Gomera (Char	0,113	0,114	0,113	0,113	0,113	0,113	0,113	0,113	0,000	0,000	0,000	0,000	0,000	0,000	0,000	0,000	0,000	0,000	0,000	0,000	0,000	0,000	0,000	0,000	0,000	0,000	0,000	0,000	0,000	0,000	0,000	0,000	0,000																							
	G. caribaeus																																						*G. caribaeus* (Acc.No. AB908139)																					
33	PL6B3	MK649672	La Gomera (Santi	0,116	0,117	0,116	0,116	0,116	0,116	0,116	0,116	0,100	0,100	0,100	0,100	0,100	0,100	0,100	0,100	0,100	0,100	0,100	0,100	0,100	0,100	0,100	0,100	0,100	0,100	0,100	0,100	0,100	0,100	0,100	0,100	0,100	0,100	0,001																					
34	PL6C3	MK649670	La Gomera (Santi	0,116	0,117	0,116	0,116	0,116	0,116	0,116	0,116	0,100	0,100	0,100	0,100	0,100	0,100	0,100	0,100	0,100	0,100	0,100	0,100	0,100	0,100	0,100	0,100	0,100	0,100	0,100	0,100	0,100	0,100	0,100	0,100	0,100	0,100	0,001	0,000																				
35	PL6D4	MK649671	La Gomera (Santi	0,116	0,117	0,116	0,116	0,116	0,116	0,116	0,116	0,100	0,100	0,100	0,100	0,100	0,100	0,100	0,100	0,100	0,100	0,100	0,100	0,100	0,100	0,100	0,100	0,100	0,100	0,100	0,100	0,100	0,100	0,100	0,100	0,100	0,100	0,001	0,000	0,000																			
36	PL11E7	MK649652	La Gomera (Santi	0,116	0,117	0,116	0,116	0,116	0,116	0,116	0,116	0,100	0,100	0,100	0,100	0,100	0,100	0,100	0,100	0,100	0,100	0,100	0,100	0,100	0,100	0,100	0,100	0,100	0,100	0,100	0,100	0,100	0,100	0,100	0,100	0,100	0,100	0,001	0,000	0,000	0,000																		
37	PL12B6	MK649658	La Gomera (Santi	0,116	0,117	0,116	0,116	0,116	0,116	0,116	0,116	0,100	0,100	0,100	0,100	0,100	0,100	0,100	0,100	0,100	0,100	0,100	0,100	0,100	0,100	0,100	0,100	0,100	0,100	0,100	0,100	0,100	0,100	0,100	0,100	0,100	0,100	0,001	0,000	0,000	0,000	0,000																	
38	PL12C2	MK649665	La Gomera (Santi	0,116	0,117	0,116	0,116	0,116	0,116	0,116	0,116	0,100	0,100	0,100	0,100	0,100	0,100	0,100	0,100	0,100	0,100	0,100	0,100	0,100	0,100	0,100	0,100	0,100	0,100	0,100	0,100	0,100	0,100	0,100	0,100	0,100	0,100	0,001	0,000	0,000	0,000	0,000	0,000																
39	PL12C3	MK649653	La Gomera (Santi	0,116	0,117	0,116	0,116	0,116	0,116	0,116	0,116	0,100	0,100	0,100	0,100	0,100	0,100	0,100	0,100	0,100	0,100	0,100	0,100	0,100	0,100	0,100	0,100	0,100	0,100	0,100	0,100	0,100	0,100	0,100	0,100	0,100	0,100	0,001	0,000	0,000	0,000	0,000	0,000	0,000															
40	PL12C4	MK649657	La Gomera (Santi	0,116	0,117	0,116	0,116	0,116	0,116	0,116	0,116	0,100	0,100	0,100	0,100	0,100	0,100	0,100	0,100	0,100	0,100	0,100	0,100	0,100	0,100	0,100	0,100	0,100	0,100	0,100	0,100	0,100	0,100	0,100	0,100	0,100	0,100	0,001	0,000	0,000	0,000	0,000	0,000	0,000	0,000														
41	PL12D5	MK649668	La Gomera (Santi	0,116	0,117	0,116	0,116	0,116	0,116	0,116	0,116	0,100	0,100	0,100	0,100	0,100	0,100	0,100	0,100	0,100	0,100	0,100	0,100	0,100	0,100	0,100	0,100	0,100	0,100	0,100	0,100	0,100	0,100	0,100	0,100	0,100	0,100	0,001	0,000	0,000	0,000	0,000	0,000	0,000	0,000	0,000													
42	PL12D6	MK649662	La Gomera (Santi	0,116	0,117	0,116	0,116	0,116	0,116	0,116	0,116	0,100	0,100	0,100	0,100	0,100	0,100	0,100	0,100	0,100	0,100	0,100	0,100	0,100	0,100	0,100	0,100	0,100	0,100	0,100	0,100	0,100	0,100	0,100	0,100	0,100	0,100	0,001	0,000	0,000	0,000	0,000	0,000	0,000	0,000	0,000	0,000												
43	PL16C5	MK649667	La Gomera (Santi	0,116	0,117	0,116	0,116	0,116	0,116	0,116	0,116	0,100	0,100	0,100	0,100	0,100	0,100	0,100	0,100	0,100	0,100	0,100	0,100	0,100	0,100	0,100	0,100	0,100	0,100	0,100	0,100	0,100	0,100	0,100	0,100	0,100	0,100	0,001	0,000	0,000	0,000	0,000	0,000	0,000	0,000	0,000	0,000	0,000											
44	PL12B4	MK649669	La Gomera (Santi	0,116	0,117	0,116	0,116	0,116	0,116	0,116	0,116	0,100	0,100	0,100	0,100	0,100	0,100	0,100	0,100	0,100	0,100	0,100	0,100	0,100	0,100	0,100	0,100	0,100	0,100	0,100	0,100	0,100	0,100	0,100	0,100	0,100	0,100	0,001	0,000	0,000	0,000	0,000	0,000	0,000	0,000	0,000	0,000	0,000	0,000										
45	**VGO1362**	MK649661	La Gomera (Santi	0,116	0,117	0,116	0,116	0,116	0,116	0,116	0,116	0,100	0,100	0,100	0,100	0,100	0,100	0,100	0,100	0,100	0,100	0,100	0,100	0,100	0,100	0,100	0,100	0,100	0,100	0,100	0,100	0,100	0,100	0,100	0,100	0,100	0,100	0,001	0,000	0,000	0,000	0,000	0,000	0,000	0,000	0,000	0,000	0,000	0,000	0,000									
46	**VGO1364**	MK649659	La Gomera (Santi	0,116	0,117	0,116	0,116	0,116	0,116	0,116	0,116	0,100	0,100	0,100	0,100	0,100	0,100	0,100	0,100	0,100	0,100	0,100	0,100	0,100	0,100	0,100	0,100	0,100	0,100	0,100	0,100	0,100	0,100	0,100	0,100	0,100	0,100	0,001	0,000	0,000	0,000	0,000	0,000	0,000	0,000	0,000	0,000	0,000	0,000	0,000	0,000								
47	**VGO1366**	MK649660	La Gomera (Santi	0,116	0,117	0,116	0,116	0,116	0,116	0,116	0,116	0,100	0,100	0,100	0,100	0,100	0,100	0,100	0,100	0,100	0,100	0,100	0,100	0,100	0,100	0,100	0,100	0,100	0,100	0,100	0,100	0,100	0,100	0,100	0,100	0,100	0,100	0,001	0,000	0,000	0,000	0,000	0,000	0,000	0,000	0,000	0,000	0,000	0,000	0,000	0,000	0,000							
48	**VGO1367**	MK649651	La Gomera (La C	0,116	0,117	0,116	0,116	0,116	0,116	0,116	0,116	0,100	0,100	0,100	0,100	0,100	0,100	0,100	0,100	0,100	0,100	0,100	0,100	0,100	0,100	0,100	0,100	0,100	0,100	0,100	0,100	0,100	0,100	0,100	0,100	0,100	0,100	0,001	0,000	0,000	0,000	0,000	0,000	0,000	0,000	0,000	0,000	0,000	0,000	0,000	0,000	0,000	0,000						
49	**VGO1369**	MK649666	La Gomera (Santi	0,116	0,117	0,116	0,116	0,116	0,116	0,116	0,116	0,100	0,100	0,100	0,100	0,100	0,100	0,100	0,100	0,100	0,100	0,100	0,100	0,100	0,100	0,100	0,100	0,100	0,100	0,100	0,100	0,100	0,100	0,100	0,100	0,100	0,100	0,001	0,000	0,000	0,000	0,000	0,000	0,000	0,000	0,000	0,000	0,000	0,000	0,000	0,000	0,000	0,000	0,000					
50	**VGO1370**	MK649673	La Gomera (Santi	0,116	0,117	0,116	0,116	0,116	0,116	0,116	0,116	0,100	0,100	0,100	0,100	0,100	0,100	0,100	0,100	0,100	0,100	0,100	0,100	0,100	0,100	0,100	0,100	0,100	0,100	0,100	0,100	0,100	0,100	0,100	0,100	0,100	0,100	0,001	0,000	0,000	0,000	0,000	0,000	0,000	0,000	0,000	0,000	0,000	0,000	0,000	0,000	0,000	0,000	0,000	0,000				
51	**VGO1371**	MK649664	La Gomera (Santi	0,116	0,117	0,116	0,116	0,116	0,116	0,116	0,116	0,100	0,100	0,100	0,100	0,100	0,100	0,100	0,100	0,100	0,100	0,100	0,100	0,100	0,100	0,100	0,100	0,100	0,100	0,100	0,100	0,100	0,100	0,100	0,100	0,100	0,100	0,001	0,000	0,000	0,000	0,000	0,000	0,000	0,000	0,000	0,000	0,000	0,000	0,000	0,000	0,000	0,000	0,000	0,000	0,000			
52	**VGO1377**	MK649663	La Gomera (Santi	0,116	0,117	0,116	0,116	0,116	0,116	0,116	0,116	0,100	0,100	0,100	0,100	0,100	0,100	0,100	0,100	0,100	0,100	0,100	0,100	0,100	0,100	0,100	0,100	0,100	0,100	0,100	0,100	0,100	0,100	0,100	0,100	0,100	0,100	0,001	0,000	0,000	0,000	0,000	0,000	0,000	0,000	0,000	0,000	0,000	0,000	0,000	0,000	0,000	0,000	0,000	0,000	0,000	0,000		
53	**VGO1382**	MK649650	La Gomera (Santi	0,116	0,117	0,116	0,116	0,116	0,116	0,116	0,116	0,100	0,100	0,100	0,100	0,100	0,100	0,100	0,100	0,100	0,100	0,100	0,100	0,100	0,100	0,100	0,100	0,100	0,100	0,100	0,100	0,100	0,100	0,100	0,100	0,100	0,100	0,001	0,000	0,000	0,000	0,000	0,000	0,000	0,000	0,000	0,000	0,000	0,000	0,000	0,000	0,000	0,000	0,000	0,000	0,000	0,000	0,000	
	G. australes																																																										*australes* (Acc.No. MG70812
54	**VGO1360**	MK649674	La Gomera (Char	0,102	0,103	0,102	0,102	0,102	0,102	0,102	0,102	0,074	0,074	0,074	0,074	0,074	0,074	0,074	0,074	0,074	0,074	0,074	0,074	0,074	0,074	0,074	0,074	0,074	0,074	0,074	0,074	0,074	0,074	0,074	0,074	0,074	0,074	0,095	0,095	0,095	0,095	0,095	0,095	0,095	0,095	0,095	0,095	0,095	0,095	0,095	0,095	0,095	0,095	0,095	0,095	0,095	0,095	0,095	0,095	0,000

	G.	*tricus* (Acc.No. J
55	PL9B6 (n=647)	0,000
56	PL9D5 (n=602)	0,000
57	PL11C4 (n=169)	0,000
58	**VGO1356** (n=604)	0,000

	G.	*G. caribaeus* (Acc.No. AB908139)
59	PL11D8 (n=765)	0,000
60	PL11E6 (n=367)	0,000
61	**VGO1365** (n=272)	0,000

Table S4. Culture strains and geographical origin of the *Gambierdiscus* species used in the morphological study.

**Species**	**Strain**	**Geographical origin**
G. australes	VGO1198	Las Américas beach, Tenerife
	VGO1263	Punta Hidalgo, Tenerife
	VGO1360	Charco Condesa Condesa, La Gomera
G. caribaeus	VGO1362	Santiago beach, La Gomera
	VGO1237	La Estaca, El Hierro
	VGO1369	Santiago beach, La Gomera
G. carolinianus	VGO1197	Alcalá, Tenerife
G. excentricus	VGO1359	Charco Condesa Condesa, La Gomera
	VGO791	Punta Hidalgo, Tenerife
	VGO1373	La Zamora beach, La Palma
G. silvae	VGO 1378	Charco Condesa Condesa, La Gomera
	VGO1180	Punta Hidalgo, Tenerife
	VGO1363	Charco Condesa Condesa, La Gomera
	VGO 1355	Charco Condesa Condesa, La Gomera
